# Irisin Is Target of Sphingosine-1-Phosphate/Sphingosine-1-Phosphate Receptor-Mediated Signaling in Skeletal Muscle Cells

**DOI:** 10.3390/ijms241310548

**Published:** 2023-06-23

**Authors:** Federica Pierucci, Antony Chirco, Elisabetta Meacci

**Affiliations:** Department of Experimental and Clinical Biomedical Sciences “Mario Serio”, University of Florence, 50134 Firenze, Italy; federica.pierucci@unifi.it (F.P.); antony.chirco@unifi.it (A.C.)

**Keywords:** irisin, myokines, sphingosine-1-phosphate, C2C12 skeletal muscle cells, sphingosine-1-phosphate receptors, skeletal muscle, sphingolipids

## Abstract

Irisin is a hormone-like myokine produced in abundance by skeletal muscle (SkM) in response to exercise. This myokine, identical in humans and mice, is involved in many signaling pathways related to metabolic processes. Despite much evidence on the regulators of irisin and the relevance of sphingolipids for SkM cell biology, the contribution of these latter bioactive lipids to the modulation of the myokine in SkM is missing. In particular, we have examined the potential involvement in irisin formation/release of sphingosine-1-phosphate (S1P), an interesting bioactive molecule able to act as an intracellular lipid mediator as well as a ligand of specific G-protein-coupled receptors (S1PR). We demonstrate the existence of distinct intracellular pools of S1P able to affect the expression of the irisin precursor FNDC. In addition, we establish the crucial role of the S1P/S1PR axis in irisin formation/release as well as the autocrine/paracrine effects of irisin on myoblast proliferation and myogenic differentiation. Altogether, these findings provide the first evidence for a functional crosstalk between the S1P/S1PR axis and irisin signaling, which may open new windows for potential therapeutic treatment of SkM dysfunctions.

## 1. Introduction

Cytokines and other peptides, named myokines, are produced and released by skeletal muscle (SkM) fibers. They exert either autocrine, paracrine, or endocrine effects [1,2,3]. Among them, irisin, first described in 2012 by Boström et al. [4] as a peroxisome proliferator-activated receptor gamma coactivator 1-α (PGC1-α)-dependent myokine, is secreted by SkM cells through proteolytic cleavage of the transmembrane protein fibronectin type III domain-containing 5 (FNDC5) expressed in the membrane [2,4,5]. One of the most important functions of this hormone-like molecule is to drive the conversion from white-fat adipocytes to brown-fat adipocytes [4]. It has also been suggested that irisin can be utilized as a potential agent for diabetes management because it ameliorates blood glucose control [6]. Thus, irisin could have beneficial effects in the treatment of obesity and metabolic disorders. Moreover, it is well documented that physical activity and contracting SkM promote an enhanced secretion of myokines, including irisin, which not only plays multiple therapeutic roles on adiposebsc tissue and SkM but also exerts beneficial endocrine effects on other organs, such as the nervous system, bone, liver, pancreatic cells, and heart [7,8,9,10]. An increasing number of studies have reported that irisin is not only correlated to metabolic diseases but also to non-metabolic diseases, including sarcopenia [11,12], and it may mediate some beneficial impacts of exercise in humans [13]. Thus, irisin may be an ideal therapeutic target for metabolic and non-metabolic chronic diseases.

Regarding SkM, irisin influences mitochondrial functions by regulating the expression of its precursor, FNDC5, through an autocrine mechanism of action and promoting the SkM phenotype [14,15]. In particular, in in vitro studies, it has been observed that irisin influences the activation/proliferation of satellite cells and myoblasts, the mononucleate precursors, and promotes the expression of pro-myogenic genes, enhancing myoblast fusion and contributing to muscle growth [16,17,18]. Following SkM injury promoted by hind-limb suspension or muscular dystrophy, irisin can improve regeneration and rescue the loss of SkM mass [14,18]. A protective effect against cell atrophy has also been demonstrated in cell cultures of C2C12 myotubes treated with the glucocorticoid dexamethasone [19].

Sphingolipids (SLs) represent the most conspicuous components in cell membranes, and, in addition to their structural functions, they also play an important role in cell signaling/responses in many cell types, including SkM cells [20,21,22,23,24,25].

The most important metabolite is sphingosine-1-phosphate (S1P), a bioactive sphingolipid that is formed from sphingosine (Sph) by two sphingosine kinase isoforms (SphK1 and SphK2). Thus, SphK plays a critical role in keeping the balance between the unphosphorylated forms of Sph, which are usually involved in cell death, and the phosphorylated forms of S1P, which are reported to act as prosurvival factors in several tissues, including SkM [26,27,28]. In particular, our group and others have shown that S1P, through the activation of its specific G-protein-coupled receptors, named S1PR1-5 [29,30] is able to act as a trophic and morphogenic factor in SkM cells [31,32,33,34] by triggering several signaling pathways strictly correlated with cell protection (i.e., phospholipase D and Akt) [35], cytoskeletal remodeling [36], and cell communication (i.e., gap junctional protein connexin-43 and the transient receptor potential cation channel regulation [32,37,38,39]. In vivo studies also pointed out that some physiological properties of SkM (i.e., muscle contractility and fatigue), as well as pathological conditions (i.e., muscle regeneration in injured mdx mice), are under the control of S1P signaling [40,41]. Recently, our group demonstrated the functional role of S1P/SphK as well as the ceramide-1-phosphate/ceramide kinase axis in the control of SkM mass wasting progression in cachectic mice and in C2C12 myotubes treated with the glucocorticoid dexamethasone (Dexa) [31,41]. S1P has a very particular mechanism of action: it can act as an intracellular mediator as well as a ligand for specific S1P receptors [5,29,34,42,43,44]. Notably, they undergo a shift in the expression pattern during cell differentiation and degeneration [31]. In particular, S1PR3, the most prevalent subtype in terminally differentiated myotubes [44], plays a role in cell structure/shape, influencing cytoskeleton remodeling and cell adhesion [45] through the RhoA/Rho-associated kinase system.

Despite all this evidence, the contribution of bioactive lipids in the regulation of myokine formation/secretion, in particular irisin, in SkM cells remains poorly investigated.

Here, we have reported that the SphK/S1P/S1PRs axis plays a role in the modulation of FNDC5 expression and irisin release/functions in C2C12 muscle cells. Notably, we also show that irisin modulates S1PR subtype expression patterns, suggesting a crosstalk between S1P/S1PR signaling and myokine functions.

## 2. Results

### 2.1. Effect of S1P Metabolism on Irisin Release and FNDC5 Expression in C2C12 Terminally Differentiated Myotubes

In order to investigate the potential functional relationship between the bioactive lipid S1P and SkM-released irisin, the conditioned medium (CM) obtained from C2C12 terminally differentiated myotubes was used. In particular, C2C12 myotubes were incubated with either exogenous S1P (1 μM) or in the presence of specific inhibitors of S1P metabolism: THI (6.5 μM), a rather specific inhibitor of S1P lyase [46], SKI-II (iSK, 5 μM), PF543 (5 μM) [47,48] or ABC294640 (10 μM) [49,50].

**Figure 1:**
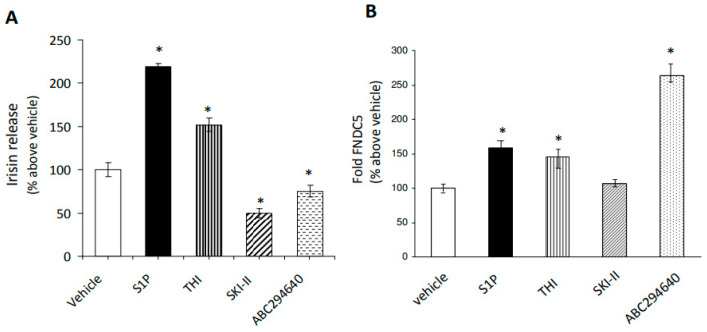
Effect of S1P accumulation on irisin release by C2C12 myotubes. (**A**) Irisin release. Irisin content was determined by ELISA assay in the conditioned media obtained from C2C12 myotubes treated for 24 h in the absence (vehicle, 0.02% DMSO) and/or in the presence of exogenous sphingosine-1-phosphate (S1P, 1 μM), S1P lyase inhibitor (THI, 6.5 μM), SphK1 inhibitor, SKI-II inhibitor (iSK, 5 μM), and SphK2 inhibitor (ABC294640, 10 μM). Relative OD_450nm_ was measured as in Methods. Data (mean ± SEM of three independent experiments performed in duplicate) are reported in the graph as percentage relative to untreated cells (vehicle) set at 100. Student’s *t*-test, * *p* < 0.05 vs. untreated cells (vehicle); (**B**) expression levels of irisin precursor, fibronectin type III domain-containing 5 (FNDC5), in C2C12 myotubes. Total RNA was obtained from C2C12 myotubes treated for 24 h in the absence (vehicle, 0.02% DMSO) and/or in the presence of S1P (1 μM), S1P lyase inhibitor (THI, 6.5 μM), SphK1 inhibitor SKI-II (iSK, 5 μM), SphK2 inhibitor (ABC294640, 10 μM). Total RNA was purified, reverse transcribed, and real-time PCR was performed using specific forward and reverse primers for FNDC5 as described in Methods. Data presented as fold change (mean ± SEM of at least 3 independent experiments) are reported in the table as percentage relative to untreated cells (vehicle) set at 100. Student’s *t*-test, * *p* < 0.05 vs. untreated cells (vehicle).

As reported in Figure 1A, the addition of exogenous S1P to the cell medium promoted a significant increase in irisin secretion (approximately 120%), and a similar effect was observed after treating C2C12 myotubes with THI. On the contrary, the inhibition of SphK1 (Figure 1A) in the presence of PF543 (41 ± 0.2%, *n* = 3) and SphK2 significantly reduced the myokine content in the culture medium of C2C12 myotubes compared to untreated cells. These findings provide the first evidence that exogenous and intracellular-formed S1P can act as novel regulators of irisin secretion.

In accordance with these results, we evaluated the potential involvement of S1P in the control of the expression level of the transmembrane protein FNDC5, the cellular precursor of irisin (Figure 1B). In view of these results, quantification by real-time PCR indicated a significant and specific increase in FNDC5 mRNA in C2C12 myotubes treated for 24 h with exogenous S1P, THI, and the SphK2 inhibitor ABC294640 or THI, whereas no change in FNDC5 expression was observed in the presence of SphK1 inhibitors, SKI-II or PF543 (108 ± 5%, *n* = 3). Taking into consideration the different intracellular localization of the two SphK isoforms, these data suggest a role for a specific pool of S1P formed by SphK2 activity in the control of FNDC5 expression at the nuclear and/or mitochondrial levels. Interestingly, in C2C12 myotubes treated with THI inhibitor, the level of FNDC5 mRNA was surprisingly higher than that of untreated cells, indicating that reduced S1P degradation also contributes to FNDC5 mRNA expression.

### 2.2. S1P Modulates Irisin-Induced Cell Proliferation and Myogenic Differentiation

**Figure 2:**
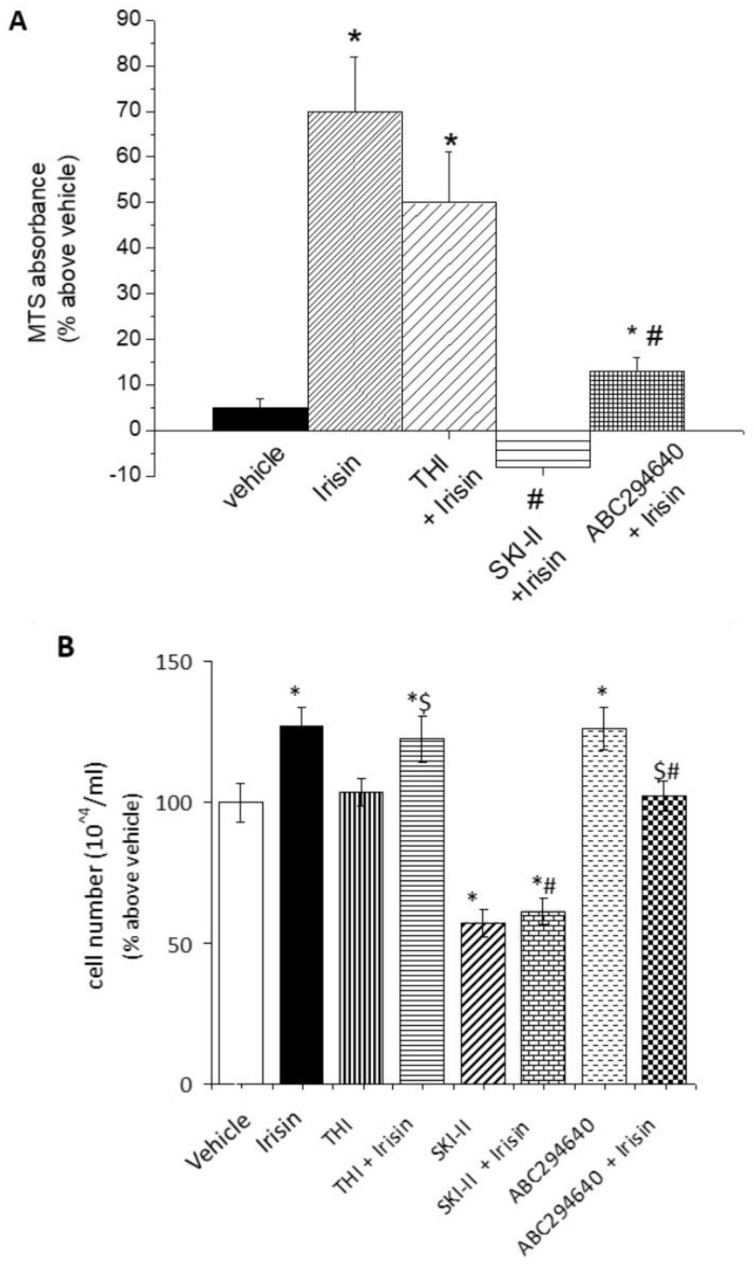
Myoblast proliferation induced by irisin requires S1P-Lyase and SphK1/2 activity. (**A**) Cell viability. C2C12 myoblasts were treated for 24 h in the absence (vehicle 0.02% DMSO) and/or in the presence of irisin (250 ng), inhibitor of S1P lyase (THI, 6.5 μM), SphK1 inhibitor SKI-II (iSK, 2 μM), and SphK2 inhibitor (ABC294640, 10 μM). An MTS-dye reduction assay was performed as described in Methods. Data (means ± S.E.M of three independent experiments performed in triplicate) are reported in the graph as percentage relative to untreated cells (vehicle). Student’s *t*-test, * *p* < 0.05 vs. untreated cells (vehicle); # *p* < 0.05 vs. irisin; (**B**) Cell proliferation. Cell counting of C2C12 myoblasts treated for 24 h in the absence (vehicle, 0.02% DMSO) and/or in the presence of irisin (250 ng), inhibitor of S1P lyase (THI, 6.5 μM), SphK1 inhibitor SKI-II (iSK, 2 μM), and selective SphK2 inhibitor (ABC294640, 10 μM). Cells were collected by trypsinization and counted using TALI^®^ cytometer as reported in Methods. Data (means ± SEM of at least three independent experiments performed in quadruplicate) are reported in the graph as percentage relative to untreated cells (vehicle) set at 100. One-way ANOVA test, * *p* < 0.05 vs. vehicle; # *p* < 0.05 vs. irisin; $ *p* < 0.05 vs. specific inhibitor THI or ABC294640.

Numerous studies have demonstrated the critical role of both irisin and S1P in the control of satellite cells and myoblast precursor proliferation [16,17,18,20,21,22,32,51,52]. Then, we analyzed whether S1P could mediate the biological functions of irisin in this cell system. As expected, the addition of recombinant irisin to the cell medium significantly increased the metabolic activity of C2C12 myoblasts compared to untreated cells (Figure 2A). Of note, this effect was significantly reduced in the presence of specific inhibitors of SphK isoforms as well as S1P lyase (Figure 2A), indicating a role for S1P or its metabolites in irisin action. Similarly, the inhibition of S1P formation, catalyzed by both SphK1 and SphK2, significantly prevented irisin-induced myoblast proliferation (Figure 2B). Interestingly, at the basal level, SphK1, but not SphK2 inhibition alone, significantly reduced cell growth, strongly supporting the role of distinct intracellular S1P pools in the regulation of myoblast proliferation. Notably, in these experimental conditions, S1P lyase inhibition was ineffective, suggesting a limited role of the S1P degradation products in this biological process.

Another interesting biological function of irisin in SkM cells is the promotion of myogenesis [15]. Therefore, in order to evaluate the potential contribution of S1P on myokine action in the early step of myogenesis, confluent cultures of C2C12 myoblasts were incubated with low serum (2% horse serum) and treated with irisin in the presence or absence of SphK inhibitors for 24 h. 

**Figure 3:**
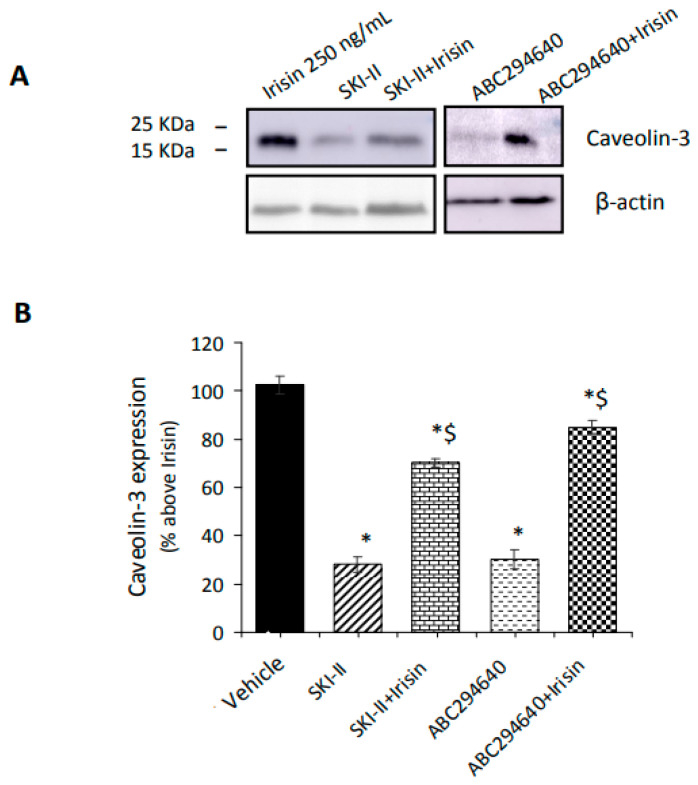
Effect of SphK activity inhibition on myogenic marker caveolin-3 expression induced by irisin. Confluent C2C12 cells were differentiated in differentiation medium containing 0.5% horse serum for 5 days and then treated for 24 h in the absence and/or presence of irisin (250 ng), SphK1 inhibitor SKI-II (iSK, 5 μM), and SphK2 inhibitor (ABC294640, 10 μM). Cell lysates (40 μg) were subjected to SDS-PAGE and immunoblotted with specific anti-caveolin-3 antibody. A blot representative of three independent experiments in triplicate (**A**) and densitometric analysis of caveolin-3 bands (**B**) are shown. Data (mean ± SEM), normalized to the β-actin band, are reported in the graph as percentage relative to irisin set at 100. One-way ANOVA test: * *p* < 0.05 vs. irisin alone; $ *p* < 0.05 vs. specific inhibitor SKI-II or ABC294640.

As reported in Figure 3A,B, in the presence of each specific SphK isoform inhibitor, the effect of irisin on the expression of the early myogenic marker caveolin-3 was significantly reduced by approximately 30% and 20%, respectively [14]. These findings support the relevance of S1P formation in the promotion of myogenesis elicited by irisin. 

**Figure 4:**
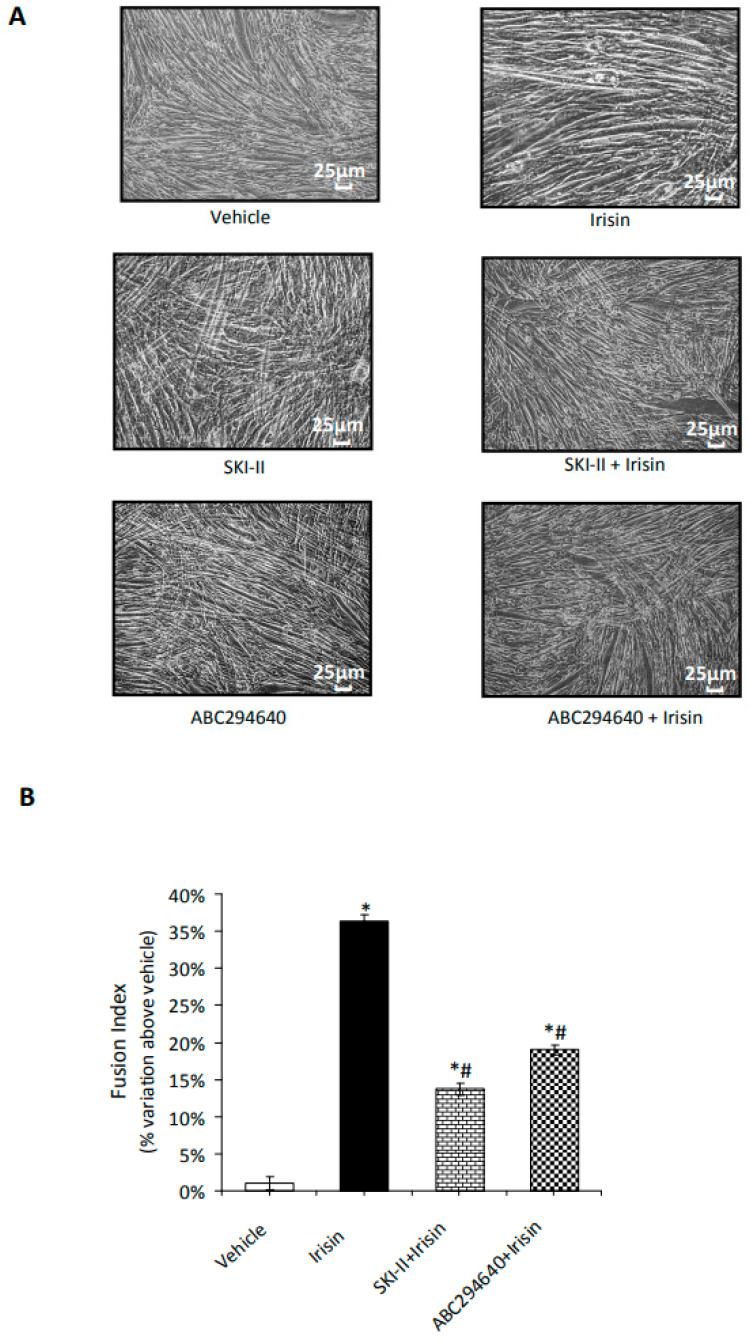
SphK activity is required for cell morphology and fusion index changes induced by irisin in C2C12 myotubes. (**A**) C2C12 cells were placed in differentiation medium, and complete myogenic differentiation was observed after 5 days of incubation. C2C12 terminally differentiated myotubes were treated as in Figure 3. Images representative of at least three independent experiments are shown. Scale bar: 25 μm. (**B**) Fusion index, determined in the different experimental conditions as reported in Figure 3, was calculated as the number of nuclei inside multinucleated cells divided by the total number of nuclei present in a field of view. The images were analyzed using the ImageJ software. Values (means ± SEM) were obtained by averaging data from the measurements of approximately 5 fields in four randomly selected regions of a well from at least 3. Data are reported in the graph as percentage relative to untreated cells (vehicle). Student’s *t*-test: * *p* < 0.05 vs. untreated cells (vehicle); # *p* < 0.05 vs. irisin.

Next, C2C12 myoblasts were induced to differentiate over a longer period of time in low serum conditions. The size and length of the myotubes increased gradually for five days. Each replicate well was photographed in four randomly selected regions (Figure 4A), the images were analyzed using the ImageJ 1.38 software, and the cell fusion index (FI) was determined (Figure 4B).

**Figure 5:**
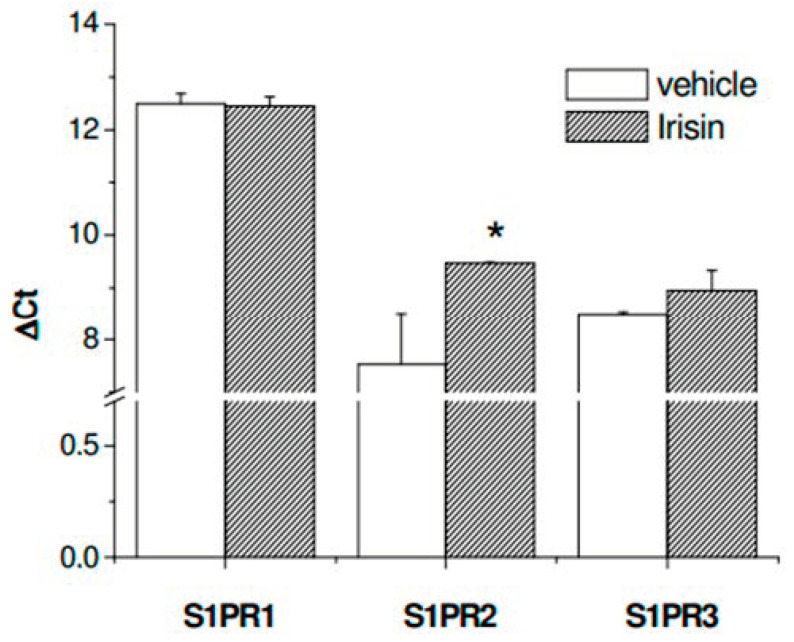
Effect of irisin on the expression levels of S1P receptor subtypes in C2C12 myotubes. Total RNA was obtained from C2C12 cells treated for 24 h in the absence (vehicle, 0.02% DMSO) and/or in the presence of irisin (250 ng). Total RNA was purified, reverse transcribed, and real-time PCR was performed using specific forward and reverse primers for each receptor subtype, as described in Methods. Data are presented as ΔCt values (mean ± SEM) of at least 3 independent experiments performed in duplicate. Student’s *t*-test, * *p* < 0.05 vs. untreated cells (vehicle).

As expected, irisin caused an increase in FI of approximately 35% compared to untreated cells, while in combination with each specific SphK isoform inhibitor, irisin action was significantly prevented, providing further evidence of the relevance of S1P in the progression of the myogenic program promoted by the myokine. Since our group previously showed a specific change in the expression pattern of receptor subtypes specific for S1P (S1PRs) in myogenic differentiation [21,31], we speculated on the potential role of irisin in the modulation of the S1PR expression pattern. As reported in Figure 5, the level of S1PR2 mRNA was strongly reduced after irisin treatment, while only a slight but not significant change was observed in S1PR1 and S1PR3 mRNA expression, indicating the existence of a potential functional crosstalk between irisin and S1PR2-mediated signaling in the control of myogenesis.

Another interesting observation is the relevance of S1P synthesis to the ability of irisin to regulate the release of the pro-inflammatory cytokine interleukin-6 (IL-6). In fact, when IL-6 content was determined in the conditioned medium of myotubes incubated with irisin in the presence or absence of SphK1 and SphK2 inhibitors, the irisin effect was significantly and specifically prevented by SphK2 (Ct value: vehicle 22.4 ± 0.17; irisin 17.9 ± 0.2 *; irisin + SKI-II 21 ± 0.12 $; irisin + ABC294640 20.8 ± 0.15 $, *n* = 3 *p* < 0.05 vs. vehicle; $ *p* < 0.05 vs. irisin alone), indicating that the anti-inflammatory role of irisin is also dependent on S1P production.

### 2.3. Irisin Release Requires S1PR-Mediated Cytoskeleton Remodeling in Myotubes 

**Figure 6:**
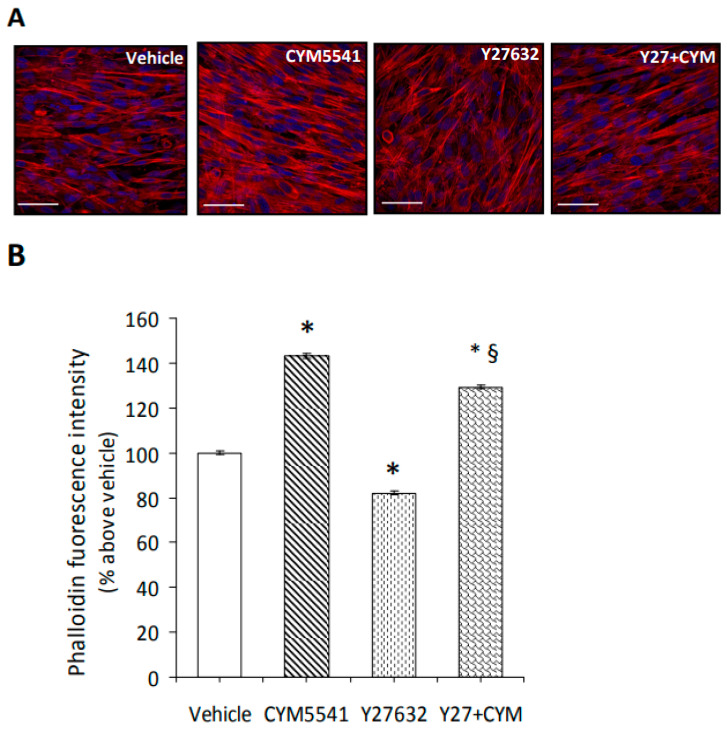
Effect of S1P/S1PR3 axis and Rho kinase on cytoskeleton remodeling in C2C12 myotubes. C2C12 myotubes were cultured for 24 h in the absence (vehicle 0.02% DMSO) and/or presence of the S1PR3 agonist (CYM5541, 2 μM) and/or Rho-associated kinase inhibitor (Y27632, 10 μM). (**A**) Phalloidin stain. C2C12 cells were cultured on glass coverslips and treated as reported above, fixed, and stained with phalloidin–tetramethylrhodamine B isothiocyanate (phalloidin–TRITC) to identify actin filaments (red). Nuclei are counterstained in blue with Dapi staining solution. Scale bar: 50 μm. The images are representative of at least three independent experiments with similar results. (**B**) Densitometric analysis. The intensity of phalloidin–TRITC signal was determined on digitized images using ImageJ software. Data (mean ± S.E.M) are reported in the graph as percentage relative to untreated cells (vehicle) set at 100. One-way ANOVA test: * *p* < 0.05 vs. untreated cells (vehicle); § *p* < 0.05 vs. Y27632.

S1PR3 is the most prevalent receptor subtype in C2C12 myotubes and SkM tissue [34,44]. Notably, as shown in Figure 6A,B, the treatment with the S1PR3 agonist, CYM5541, promotes a dynamic reorganization of the cytoskeleton, as stated by the increase in phalloidin incorporation into F-filaments running across the cell body. Moreover, the formation of well-structured F-actin filaments was significantly potentiated in the presence of CYM5541 and the inhibitor of Rho kinase, Y27632 (10 μM) [53].

These findings suggest the relevance of S1PR3-mediated signaling in Rho kinase-dependent actin remodeling in C2C12 myotubes.

**Figure 7:**
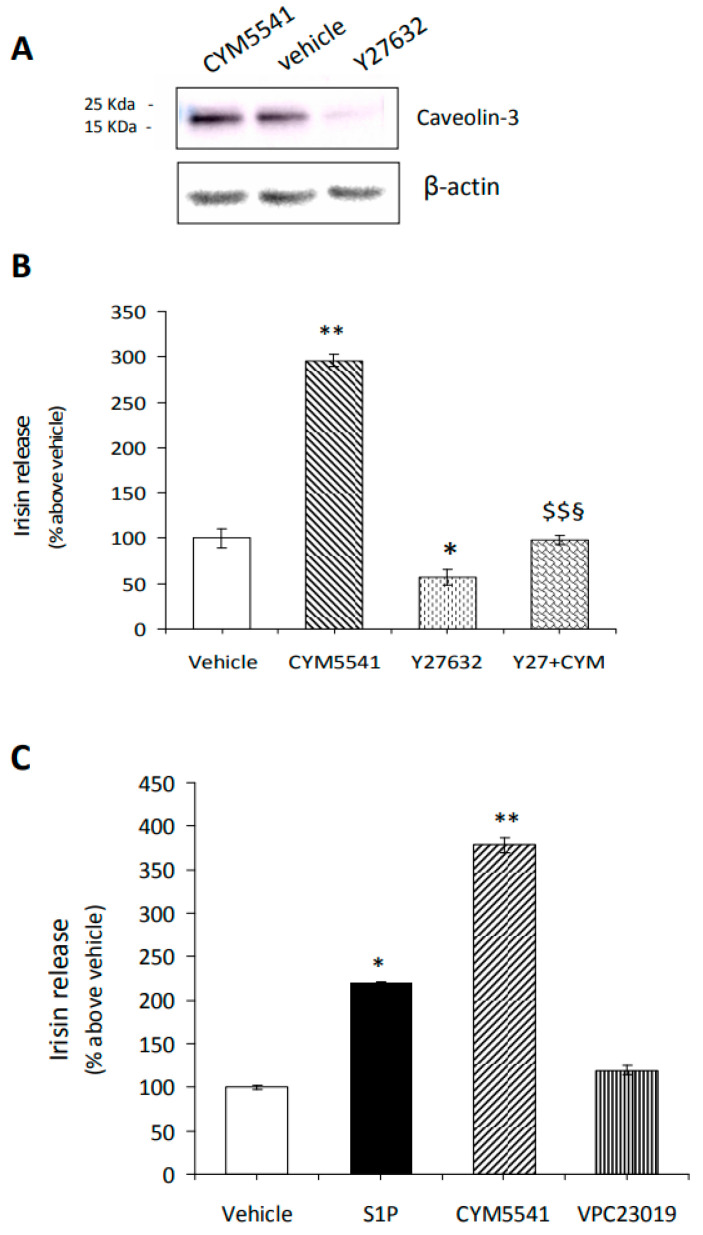
Effect of S1P/S1PR3 axis and Rho kinase inhibition on caveolin-3 expression and irisin release. (**A**) Expression of caveolin-3. Confluent C2C12 cells were differentiated as described in Figure 3 and treated for 24 h in the absence (vehicle 0.02% DMSO) and/or presence of S1PR3 agonist (CYM5541, 2 μM) and/or Rho kinase inhibitor (Y27632, 10 μM) and then processed as described in Methods. A blot representative of two independent experiments and densitometric analysis of caveolin-3 reported as relative percentage of expression on vehicle (set 100) are shown. (**B**,**C**) Irisin release. Irisin content was evaluated by ELISA assay in the conditioned media obtained from C2C12 myotubes treated for 24 h in the absence and/or presence of S1PR3 receptor agonist (CYM5541, 2 μM), and/or Rho-associated kinase inhibitor and in presence of (C) exogenous sphingosine-1-phosphate (S1P, 1 μM), S1PR3 receptor agonist (CYM5541) or S1P1/S1P3 antagonist (VPC23019, 1 μM). Relative OD_450nm_ was measured as reported in Methods. Data (mean ± SEM of three independent experiments performed in duplicate) are reported in the graph as percentage relative to untreated cells (vehicle) set at 100. One-way ANOVA test: (**B**) * *p* < 0.05; ** *p* < 0.01 vs. untreated cells (vehicle); § *p* < 0.05 vs. Y27632; $$ *p* < 0.01 vs. CYM5541. (**C**) Student’s *t*-test, * *p* < 0.05; ** *p* < 0.01 vs. untreated cells (vehicle).

In these conditions, treatment with the S1PR3 agonist and Rho-associated kinase inhibitor Y27632 has an opposite effect on the expression of the early myogenic marker caveolin-3 (Figure 7A) and on irisin release (Figure 7B). Successively, we measured the irisin concentration in the culture medium of C2C12 myotubes treated either with exogenous S1P (1 μM) or with CYM5541 (Figure 7C). 

Notably, both S1P and S1PR3 agonists were able to significantly increase irisin release and counteract the reduction in myokine secretion observed when myotubes were treated with Y27632 alone [54], supporting the relevance of S1PR3-mediated signaling for irisin release. In particular, the increase in irisin secretion, elicited by CYM5541, was higher than that obtained by treating myotubes with exogenous S1P (1 μM), suggesting a potential negative role of the other receptor subtypes (i.e., S1PR2-mediated signaling) on irisin release (Meacci, personal communication). On the other hand, VPC23019 (1 μM), a non-selective S1P1/S1P3 antagonist [55], did not significantly affect hormone secretion compared to untreated cells (Figure 7C). 

These findings demonstrate that irisin secretion depends not only on S1P synthesis but also on S1P/S1PR3-mediated signaling, which involves cytoskeletal remodeling.

### 2.4. S1P Modulates the Secretion of Irisin Promoted by Trimetazidine

**Figure 8:**
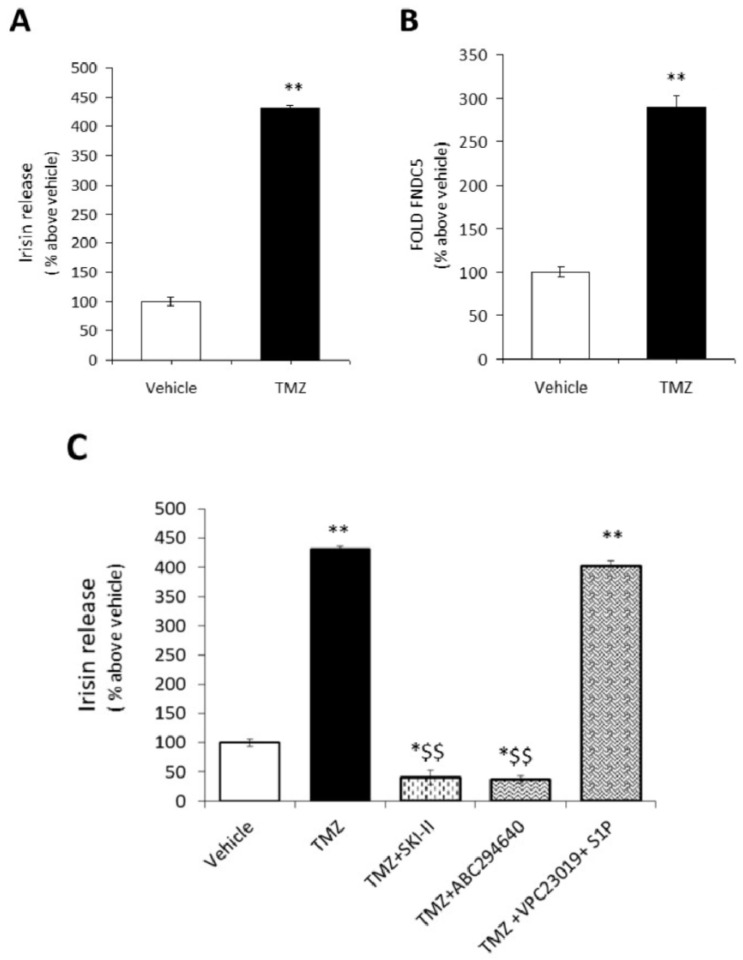
TMZ-induced irisin release requires S1P1/3-initiated signaling and SphK1/2 activity. (**A**) Irisin release. Irisin content was evaluated by ELISA assay in the conditioned media obtained from C2C12 myotubes treated for 24 h in the absence (vehicle) and/or in the presence of metabolic modulator trimetazidine (TMZ, 200 μM). Relative OD_450nm_ was measured as in Methods. Data (mean ± SEM of three independent experiments performed in duplicate) are reported in the graph as percentage relative to untreated cells (vehicle) set as 100. Student’s *t*-test, ** *p* < 0.01 vs. untreated cells (vehicle). (**B**) Expression levels of irisin precursor, fibronectin type III domain-containing 5 (FNDC5) in C2C12 myotubes. Total RNA was obtained from C2C12 myotubes treated for 24 h in the absence (vehicle 0.02% DMSO) and/or in the presence of inhibitor of trimetazidine (TMZ, 200 μM). After 24 h total RNA was purified, reverse transcribed, and real-time PCR was performed using specific forward and reverse primers for FNDC5 as described in Methods. Data are presented as fold change (mean ± SEM of at least 3 independent experiments). Data are reported as percentage relative to untreated cells (vehicle) set at 100. Student’s *t*-test, ** *p* < 0.01 vs. untreated cells (vehicle). (**C**) Irisin release. Irisin content was evaluated by ELISA assay in the conditioned media obtained from C2C12 myotubes treated for 24 h in the absence (vehicle 0.02% DMSO) and/or presence of trimetazidine (TMZ, 200 μM), SphK1 inhibitor SKI-II (iSK, 5 μM), SphK2 inhibitor (ABC294640, 10 μM), S1P1/S1P3 antagonist (VPC23019, 1 μM), exogenous sphingosine-1-phosphate (S1P, 1 μM). Relative OD_450nm_ was measured as described in Methods. Data (mean ± SEM of three independent experiments performed in duplicate) are reported in the graph as percentage relative to untreated cells (vehicle) set as 100. One-way ANOVA test: * *p* < 0.05; ** *p* < 0.01 vs. untreated cells (vehicle); $$ *p* < 0.01 vs. TMZ.

Previous reports have demonstrated that physical activity enhances the levels of myokines, and the metabolic modulator trimetazidine (TMZ) mimics exercise capacity by enhancing mitochondrial quality control [56,57]. Therefore, in order to explore whether S1P synthesis and the S1P/S1PR3 axis could be involved in TMZ-induced irisin release, an ELISA assay was performed on culture medium of terminally differentiated myotubes treated with SphK1 or SphK2 inhibitors or an S1PR1/S1PR3 receptor subtype antagonist (VPC23019) and exogenous S1P (1 μM) in the presence of TMZ (200 μM). As expected, TMZ induced a significant release of myokine and an increase in the FNDC5 mRNA level (Figure 8A,B). Notably, the inhibition of SphK1 as well as SphK2 activity significantly limits irisin secretion elicited by TMZ. On the contrary, the addition of exogenous S1P and VPC23019 together in the presence of TMZ did not induce any significant effect on irisin release compared to TMZ alone (Figure 8C). These findings suggested that irisin release promoted by TMZ required S1P formation but not S1PR3-mediated signaling.

## 3. Discussion

**Figure 9:**
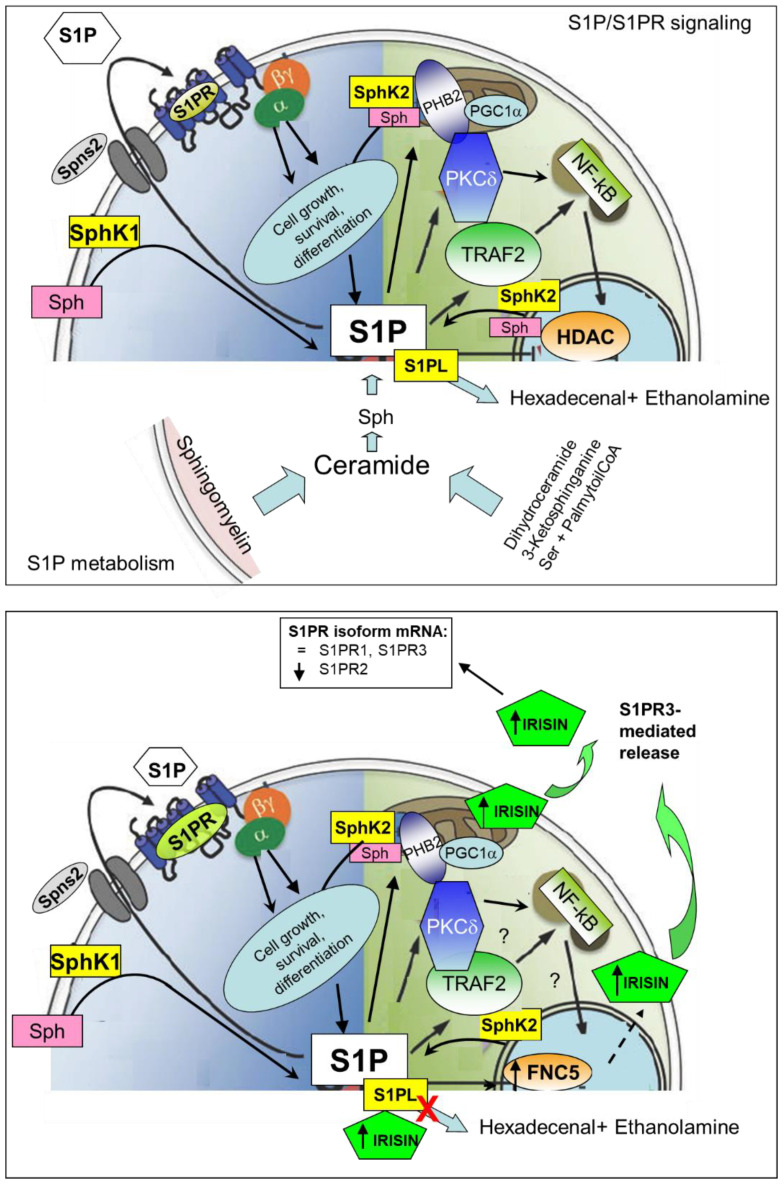
Scheme of S1P metabolism, S1P/S1PR signaling and functional crosstalk between S1P/S1PR axis and irisin in C2C12 skeletal muscle cells. S1P, sphingosine-1-phoshate; Sph, sphingosine; SphK, sphingosine kinase; S1PL, S1P lyase; S1PR, S1PR receptor; Spns2, Spinster homology 2; PHB2, prohibitin 2; PGC1α, PPAR-gamma coactivator 1-alpha; PKCδ protein kinase C-delta; TRAF2, TNF receptor-associated factor 2; NF-kB. Nuclear factor kappa-light-chain-enhancer of activated B cells. Red cross indicates the effect of S1PL (S1P lyase) inhibition. Dotted arrow indicates unknown multistep pathway.

In the current study, we have clarified the effects of the S1P/S1PR axis on irisin formation/secretion in murine SkM cells and proposed a functional crosstalk between the bioactive S1P and myokine. To our knowledge, the findings of this study represent the first evidence that in myoblasts as well as in terminally differentiated myotubes, S1P/S1PR-mediated signaling controls the formation and secretion of irisin and mediates its autocrine/paracrine action, as summarized in Figure 9.

### 3.1. Inhibition of SphK2 and S1P Lyase, but Not SphK1, Affects FNDC5 Expression in Myotubes

Notably, we demonstrate that a specifically localized pool of S1P can modulate FNDC5 mRNA expression, likely by interfering at the nuclear level with its gene expression. Indeed, it is reported that (a) S1P is formed by conserved lipid kinases, SphK1 and SphK2 isoforms, and is highly enriched in specific intracellular compartments [58,59,60,61]; (b) S1P, formed by SphK2, can directly modulate histone deacetylase activity [62]; and (c) FNDC5 gene transcription is regulated by histone H3 acetylation and methylation [63]. Therefore, the here-reported finding that the specific SphK2 inhibition significantly reduces FNDC5 gene expression may suggest a possible epigenetic regulation of FNDC5 by a specific pool of S1P and open new windows in the topic.

A further possibility is that S1P, produced in mitochondria, affects FNDC5/irisin processing. Although, in different cell systems, it has been reported that S1P directly binds and modulates the mitochondrial mitophagy protein prohibitin 2 [59]. Prohibitin 2 is able to promote mitochondrial biogenesis by activating the peroxisome proliferator-activated receptor γ coactivator 1α [60,64], a master regulator of energy metabolism and inducer of the irisin precursor FNDC5 [4].

### 3.2. Irisin Secretion Depends on S1P Accumulation and S1PR Signalling in Myotubes

Another interesting finding of this study is that irisin secretion depends on S1P accumulation and S1PR signaling. In fact, the increased secretion of this myokine occurs in THI-treated cells, in which S1P is not irreversibly degraded by S1P lyase [65], and a decreased release is found in SphK1-silenced cells, in which S1P synthesis is reduced [21].

It is well documented that physical activity and contracting SkM induce an enhanced secretion of several myokines, including irisin [66], and that a possible link between irisin secretion, exercise, and SkM protection and regeneration exists in in vivo animal models as well as in humans [67,68]. Here, we observed that the activation of SphK as well as S1PR3-mediated signaling appears to be necessary for irisin secretion. In particular, we demonstrated that exogenous S1P and S1PR3 agonists, CYM5541, significantly increased irisin release, whereas S1PR3 antagonists, VPC23019 or Ty52156 (MEACCI personal communication), strongly reduced irisin secretion, supporting a role of S1PR3-mediated signaling and cytoskeleton remodeling in the modulation of the autocrine/paracrine actions of this myokine. On the other hand, the irisin secretion promoted by TMZ, a modulator of SkM metabolism used to mimic exercise performance, was reduced by limiting S1P formation. Notably, in a recent human study, the concentration of total SL species, including S1P, was reported to be significantly enhanced by acute exercise (2 h) [69]. Increased S1P content was also found to have beneficial effects on the promotion of fatigue resistance [70].

### 3.3. Functional Crosstalk between Irisin and S1P in Skeletal Muscle Cells

Another set of experiments underlines the existence of a functional crosstalk between irisin and S1P in SkM cells. SphK1- and SphK2-mediated S1P synthesis is required for the ability of irisin to modulate myoblast growth and myogenesis. In fact, the induction of cell proliferation and the promotion of early and late myogenic markers by irisin were limited in the presence of reduced S1P synthesis. The ability of irisin to modulate IL-6 signaling [18] was also mediated by SphK/S1P. On the other hand, irisin is able to affect S1PR expression, strongly reducing the expression of S1PR2, which is known to be relevant for the promotion of myogenesis as well as to prevent cell atrophy [31]. Only small changes were observed in S1PR1 and S1PR3 expression. This effect on S1PR expression remodeling appears to be a crucial aspect worth considering in future studies to explain other biological outcomes of the myokine. We believe that the modulation of the S1P system and irisin may represent a tightly orchestrated program for allowing gene expression regulation to finalize SkM cell survival. In fact, after tissue injury (i.e., oxidative stress), SphK and S1P lyase activities have been reported to be dynamically regulated [71]. The upregulation of S1P lyase has been observed in dystrophic SkM tissue associated with a significant reduction in plasma S1P content [72,73], and a reduction in circulating S1P worsens the mdx soleus muscle dystrophic phenotype [74], leading also to an impairment of muscle regeneration and stem cell recruitment.

Furthermore, altered levels of S1P have been reported in patients, which show SkM tissue disorders correlated to SARS-CoV-2 infection [75]. Similarly, the plasma level of irisin has been found to be reduced in animal models of SkM dysfunction [72] as well as in male patients affected by myotonic dystrophy [76,77].

Regarding SkM atrophy, our group reported that SphK/S1P signaling and the ceramide kinase/ceramide-1-phosphate (C1P) axis are unpaired in in vivo cachectic mice as well as in an in vitro model of cancer cachexia, represented by dexamethasone (Dexa)-treated myotubes [19,31,42,78], suggesting a role for these bioactive lipids in preserving the SkM cell phenotype. Notably, the downregulation of S1PR1/3 that occurs in Dexa-treated myotubes as well as in cachectic mice [31] is responsible for the impairment of irisin secretion and of its function on SkM mass (Meacci et al., personal communication). Thus, both irisin and S1P may be suggested as useful tools for therapeutic treatments. On this basis, irisin treatment is reported to improve SkM mass and strength in dystrophic mdx mice [18], and an increased level of S1P is shown to help muscle regeneration in acutely injured *mdx* mice [41]. Altogether, these results provide further proof that inside-out S1P signaling is critically involved in SkM biology and provide support to the concept that the specific targeting of the S1P/S1PR axis could represent an exploitable approach to modulate in vivo irisin with beneficial effects on SkM aging/disorders.

## 4. Materials and Methods

### 4.1. Materials Biochemicals, Cell Culture Reagents

Dulbecco’s Modified Eagle’s Medium (DMEM), phosphate-buffered saline (PBS), fetal calf serum (FCS), horse serum (HS), penicillin/streptomycin, protease inhibitor cocktail, and bovine serum albumin were purchased from Sigma-Aldrich (Milan, Italy). C2C12 cells were obtained from the American Type Culture Collection (ATCC, Manassa, VA, USA); exogenous sphingosine-1-phosphate (S1P) was from Calbiochem (San Diego, CA, USA); 4-[[4-(4-chlorophenyl)-2-thiazolyl]amino]phenol Compound II, SKI-II, a selective non-lipid inhibitor of SphK1 [47], and ABC294640, SphK2 inhibitor [49], were from Tocris Bioscience (Bristol, UK). THI, inhibitor of S1P lyase [38], was obtained from Sigma-Aldrich. S1P receptor subtypes (S1PRs) agonist/antagonist: S1PR3 receptor agonist CYM5541 was from Tocris Bioscience (Bristol, UK) [79]; S1P1 and S1P3 antagonist VPC23019 was from Avanti Polar Lipids (USA) [55]. Rho-associated kinase inhibitor (Y27632) was from Abcam (Cambridge, UK) [54]. In another set of experiments, the cells were exposed to the metabolic modulator trimetazidine (TMZ) from Sigma-Aldrich [80] and to the irisin standard protein concentrate from BioVision, Inc., Life Science (Los Angeles, CA, USA). For confocal laser scanning microscope analysis, paraformaldehyde solution 4% (PFA) in PBS, Triton X-100 detergent, bovine serum albumin (BSA), gel mount, and DAPI staining solution were from Sigma-Aldrich. For real-time PCR data, TRIzol reagent, high-capacity cDNA reverse transcription kit, and SYBR Green reagent were from Life Technologies and Thermo Fisher Scientific (Carlsbad, CA, USA); the Bradford microassay for measuring protein concentration and non-fat dry milk in blocking buffer were from Bio-Rad (Hercules, CA, USA). Amersham Hybond P 0.45 PVDF membrane and chemiluminescence kit used for Western blotting analysis were from GE Healthcare (Buckinghamshire, UK); rabbit polyclonal anti-caveolin-3 (1:1000) antibody was from Genetex (Irvine, CA, USA); rabbit polyclonal anti-β-actin (1:2000) was obtained from Sigma-Aldrich; and mouse anti-rabbit (1:10,000) secondary antibody conjugated to horseradish peroxidase was from Santa Cruz Biotechnology (Santa Cruz, CA, USA). For immunofluorescence confocal images, fluorescent phallotoxin—phalloidin–tetramethylrhodamine B isothiocyanate (phalloidin–TRITC 1:400)—was from Santa Cruz Biotechnology (Santa Cruz, CA, USA). Irisin concentration was measured using an irisin-competitive ELISA kit (AdipoGen Life Sciences, Liestal, Switzerland) [81]. The CellTiter 96 Aqueous One Solution Cell Proliferation Assay Kit was from Promega (Madison, WI, USA). The Tali cytometer for cell counting was from Life Technologies (Eugene, OR, USA).

### 4.2. Cell Culture and Treatments

Murine C2C12 skeletal myoblasts obtained from the American Type Culture Collection (ATCC) were routinely grown in Dulbecco’s Modified Eagle’s Medium (DMEM) supplemented with 1% L-glutamine, 1% penicillin/streptomycin, and 10% fetal bovine serum (FBS). For myotube experiments, in which differentiation of myoblasts into myotubes was induced, cells were seeded in 6-well plates at a concentration of 0.2 × 10^6^ cells/mL. When the cells reached 90–100% confluence (48 h after seeding), the medium was replaced with differentiation medium (DM), which was composed of DMEM containing antibiotics, glutamine, and 0.5% horse serum (HS). Complete cell differentiation was observed after 5 days of incubation with 0.5% HS. Irisin concentration and expression levels of irisin precursor, fibronectin type III domain-containing 5 (FNDC5), and S1P receptor subtypes (S1PRs) by real-time PCR were evaluated in C2C12 skeletal myotubes incubated for 24 h with the following compounds: irisin standard protein concentrate (250 ng/mL); 4-[[4-(4-chlorophenyl)-2-thiazolyl]amino]phenol Compound II, SKI-II, selective non-lipid inhibitor of SphK1 2–5 μM [47], ABC294640, SphK2 inhibitor 10 μM [49]; THI, inhibitor of S1P lyase 6.5 μM [38], and metabolic modulator trimetazidine TMZ, 200 μM [80]. In another set of experiments (confocal laser scanning microscope analysis and ELISA assay for irisin detection), C2C12 myotubes were treated for 24 h with S1P receptor 3 subtype (S1PR3) agonist CYM5541 2 μM and S1P1/S1P3 antagonist VPC23019 1 μM [55]; Rho-associated kinase inhibitor (Y27632) 10 μM [54]; and exogenous sphingosine-1-phosphate (S1P) 1 μM. For cell proliferation and viability analyses, C2C12 myoblasts were seeded in 96-well plates at a concentration of 0.2 × 10^4^ cells/100 μL. Cells were counted with the Burker chamber and/or after fixation and propidium iodide staining by TALI^®^ cytometry. Finally, for real-time PCR analysis of IL-6 and for caveolin-3 expression by Western blotting, C2C12 cells were seeded in 6-well plates at a concentration of 0.25 × 10^6^ cells/mL. The cells were placed in differentiation medium (DM) with low serum content (0.5% HS) for 48 h when the culture reached 100% confluence (48 h after seeding) and were subsequently treated for 24 h with SphK1 inhibitor SKI-II (iSK, 2–5 μM) or selective SphK2 inhibitor (ABC294640, 10 μM) in the absence and/or presence of irisin standard protein concentrate (250 ng/mL). Cell lysates were collected after 72 h.

### 4.3. Enzyme-Linked Immunosorbent Assay (ELISA) Analysis of Irisin Concentration in C2C12 Myotubes Culture Medium

Irisin levels were measured using an irisin-competitive ELISA kit (AdipoGen Life Sciences) [81]. C2C12 myotubes were treated in differentiation medium as described above, and after 24 h, the conditioned medium was collected and centrifuged at 20,000× *g* for 10 min. Subsequently, 50 μL of the irisin standard solution and 50 μL of cell culture supernatants from each sample were added to wells coated with specific detection antibody. After adding 100 μL of working reagent conjugate buffer, the solution was incubated for 2 h at 37 °C. The contents of each well were removed, and the wells were washed three times with a wash buffer. Then, 100 μL of the 3,3′,5,5′-tetramethylbenzidine (TMB) substrate solution was added to each well and incubated for 30 min at 37 °C. After adding 50 μL of stop solution, an ELISA reader (Model 550 microplate reader, Bio-Rad Inc., Hercules, CA, USA) was used to measure at 450 nm absorbance within 10 min, and the measurement was entered in the standard curve for calculation. The intra- and inter-assay coefficients of variation (CV) for irisin were ≤8% and ≤12% and the detection assay range was 0.001–5 μg/mL. Irisin levels in treated cells were reported as a percentage relative to untreated cells (vehicle 0.02% DMSO) set at 100.

### 4.4. Cell Proliferation, Viability and Morphological Analyses

For cell proliferation and viability analyses, C2C12 myoblasts were seeded in 96-well plates at a concentration of 0.2 × 10^4^ cells/100 μL. When the cells reached ~60% confluence (24 h after seeding), the medium was replaced with differentiation medium (DM), and the cells were incubated for 24 h with THI, inhibitor of S1P lyase (6.5 μM), SphK1 inhibitor SKI-II (iSK, 2–5 μM), or selective SphK2 inhibitor (ABC294640, 10 μM) in the absence and/or presence of irisin standard protein concentrate (250 ng/mL). Cells were counted with the Burker chamber and/or after fixation and propidium iodide staining by TALI^®^ cytometry (Life Technologies). Cell viability was evaluated by non-radioactive cell assay (MTT) according to the manufacturer’s protocol [22,82], adding in each well 10 µL of MTS (5 mg/mL, CellTiter 96-Aqueous One Solution Cell Proliferation Assay, Promega). The absorbance was measured at 490 nm after 10, 30 min, 1 h, and 2 h of incubation with a microplate reader (Model 550 microplate reader, Bio-Rad Inc., Hercules, CA, USA).

Myoblast fusion assays were performed as previously described [83]. C2C12 myoblasts were cultured in six-well plates at a concentration of 0.2 × 10^6^ cells/mL and induced to differentiate over a period of 5 days after a change to low serum (DM) conditions (day 0). Terminally differentiated myotubes were treated for 24 h as described above. For all analyses, 5–6 images per well (i.e., experimental *n*) were taken at random sections, and the total nuclei were counted using ImageJ (National Institutes of Health, Bethesda, MD, USA). Each well was photographed in four randomly selected regions using a digital camera (Nikon Camera) adapted to an inverted microscope. For each field, the number of nuclei incorporated in myotubes and the total number of nuclei were scored. The fusion index (FI) was calculated as the percentage of total nuclei incorporated in myotubes.

### 4.5. Quantification of RNA and Expression by Quantitative Real-Time Polymerase Chain Reaction (qPCR)

Expression levels of irisin precursor, fibronectin type III domain-containing 5 (FNDC5), S1P receptor subtypes (S1P1-S1P2-S1P3), and IL-6 were quantified by real-time PCR. Specific primers for FNDC5, S1P1-S1P2-S1P3, IL-6, and the housekeeping gene GAPDH (glyceraldehyde-3 phosphate dehydrogenase) were designed using the NCBI BLAST NUCLEOTIDE program (Rockville Pike, Bethesda, MD, USA). GAPDH mRNA was the endogenous control used to normalize mRNA concentrations. Total RNA was extracted using the TRIzol reagent (Invitrogen, Waltham, MA, USA), according to the manufacturer’s instructions. The quantity and quality of the RNA extracts were measured using a Nanodrop (Thermo Scientific, Waltham, MA, USA). First-strand cDNA was synthesized in a 20 μL reverse transcription (RT) reaction with a unit of 1 μg of total RNA using the High-Capacity cDNA Reverse Transcription Kit (Life Technologies). The reaction conditions were incubated for 10 min at 25 °C, 120 min at 37 °C, and 5 min at 85 °C, and then the samples were stored at −20 °C.

The real-time PCR reaction was performed in a 25 μL volume and consisted of cDNA 100 ng, 0.25 μM of each primer (primer stocks solution 100 μM, Sigma-Aldrich), and 2X Power SYBR Green PCR Master Mix (Life Technologies). The gene amplification was conducted at MIC Diatech Pharmacogenetics (Ancona, Italy), according to these thermal conditions: 95 °C for 10 min, followed by 40 cycles of a three-step reaction; denaturation at 95 °C for 30 s, annealing at 52–60 °C for 30 s, elongation at 72 °C for 45 s (fluorescence was collected during the elongation step), followed by a melting curve from 65 to 95 °C in a 10 s increment of 0.5 °C (95 °C for 15 s, 60 °C for 60 s, 95 °C for 15 s, and 60 °C for 15 s for the dissociation analysis). The sequences of the primers used in this study are provided below:

FNDC5:

Forward: 5′-TTGCCATCTCTCAGCAGAAGA-3′

Reverse 5′-GGCCTGCACATGGACGATA-3′

S1P1:

Forward: 5′-CCGCAAGAACATCTCCAAGG-3′

Reverse: 5′-GGCAATGAAGACACTCAGGA-3′

S1P2:

Forward: 5′-CATCG TGGTGGAGAATCTTCTG-3′

Reverse: 5′-CAGGTTGCCA AGGAACAGGTA-3′

S1P3:

Forward: 5′-CCACCTG CAGCTTATGGCC-3′

Reverse: 5′-GGCAA TTAGCCAGCACATCCC-3′

IL-6:

Forward: 5′-TGAACTCCTTCTCCACAAGCG-3′

Reverse: 5′-TCTGAAGAGGTGAGTGGCTGTC-3′

GAPDH:

Forward: 5′-GGCAAATTCAACGGCACAGTC-3′

Reverse: 5′-TCGCTCCTGGAAGATGGTG-3′

Both positive and negative controls were included in each assay. Relative FNDC5 expression fold change was calculated using this method: fold change = 2−^∆∆Ct^, where ∆Ct is the difference in Ct between the target gene and the housekeeping one, and ∆∆Ct is the difference between the ∆Ct of the interest sample and the ∆Ct of the reference one. S1P1, S1P2, S1P3, and IL-6 RNA levels were reported as ΔCt values (mean ± SEM) and normalized against the expression of GAPDH.

### 4.6. Western Blot Analysis

Western blotting was performed to determine protein levels of the myogenic marker caveolin-3 in C2C12 cells. Cells were lysed at 4 °C using RIPA buffer containing 50 mM Tris–HCl, pH 7.5, 120 mM NaCl, 1 mM EDTA, 6 mM EGTA, 15 mM Na_4_P_2_O_7_, 20 mM NaF, 1% Nonidet, and protease inhibitor cocktail (1.04 mM AEBSF, 0.08 mM aprotinin, 0.02 mM leupeptin, 0.04 mM bestatin, 15 mM pepstatin A, 14 mM E-64, 2 mM Na_3_VO_4_) (Sigma-Aldrich). Subsequently, cell lysates were centrifuged at 600× *g* for 6 min at 4 °C, and protein concentration was measured using the Bradford microassay (Bio-Rad). For all Western blot analyses, aliquots containing 40 μg of proteins were diluted in 2× loading buffer (10% SDS, 50% glycerol, 0.25% bromophenol blue, and 0.25 M Tris–HCl, pH 6.8) (Life Technologies, Thermo Fisher Scientific) and reduced at 90 °C for 10 min. Samples were subjected to 15% SDS-polyacrylamide gel electrophoresis (SDS-PAGE) (Bio-Rad) and transferred by electroblotting to an Amersham Hybond P 0.45 PVDF membrane (GE Healthcare). The blots, after sufficient washes with phosphate-buffered saline PBS, were first blocked with PBST buffer (150 mM NaCl, 0.05% Tween-20, and 20 mM Tris–HCl, pH 7.4) containing 5% non-fat dry milk (Bio-Rad) for 1 h at room temperature. After blocking, the membranes were washed in PBST and then incubated overnight with the following primary antibodies: rabbit polyclonal anti-caveolin-3 (1:1000) (Genetex) and rabbit polyclonal anti-β-actin (1:2000) (Sigma-Aldrich). After sufficient washes with PBST, the blots were incubated for 1 h at room temperature with mouse anti-rabbit horseradish peroxidase-conjugated secondary antibody (Santa Cruz) at a 1:10,000 dilution. Following three washes with PBST, immunoreactive protein bands were visualized using ECL Western Blotting Detection Reagents (GE Healthcare), and, finally, capture and analysis of high resolution digital images of protein in membranes were obtained using the Amersham Imager 600 series system (GE Healthcare Life Sciences, Marlborough, MA, USA). The identity of the bands on the Western blots was confirmed by comparing them to a precision molecular weight marker (Bio-Rad). Densitometric analysis of the bands was performed using NIH IMAGE J (ImageJ software, Bethesda, MD, USA) and Quantity-One (Imaging and Analysis Software quantity-one 4.6 version by Bio-Rad Laboratories, Hercules, CA, USA). Band intensity was reported as a relative percentage (means ± SEM) obtained by calculating the ratio between target protein and β-actin. 

### 4.7. Confocal Laser Scanning Microscope Analysis

Immunofluorescence analyses on fixed C2C12 myotubes were performed as reported previously in [84]. C2C12 myotubes grown on glass coverslips 24 h after treatment were fixed with 4% PFA (Sigma-Aldrich) for 20 min at RT. After permeabilization with cold PBS solution containing 0.3% Triton (Sigma-Aldrich) for 5 min, the fixed cells were blocked with 0.3% Triton and 3% bovine serum albumin (BSA, Sigma-Aldrich) in PBS for 30 min at RT and then incubated with fluorescent phallotoxin—phalloidin–tetramethylrhodamine B isothiocyanate (phalloidin–TRITC 1:400) (Santa Cruz)—overnight at 4 °C to evaluate actin filament organization. In the same experiments, nuclei were counterstained with DAPI staining solution (DAPI, Stock solution 250 nM, Sigma-Aldrich). After washing with cold PBS solution, the coverslips containing the immuno-labeled cells were mounted with a fluoromount aqueous mounting gel mount (Sigma-Aldrich) and observed under a confocal Leica TCS SP8 microscope (Leica Microsystems, Wetzlar, Germany). Observations were performed using a Leica Plan Apo 636/1.43NA oil immersion 40× objective. A series of optical sections (1024 × 1024 pixels each; pixel size 204.3 nm) of 0.4 mm in thickness were taken through the depth of the cells at intervals of 0.4 μm. Images were then projected onto a single “extended focus” image. Densitometric analyses of the intensity of Phalloidin fluorescent signals were performed on digitized images using ImageJ software (http://rsbweb.nih.gov/ij) in 20 regions of interest (ROI) of 100 μm^2^ for each confocal stack (at least 10). Phalloidin fluorescent intensity was reported as a relative percentage (means ± SEM), obtained by calculating the ratio of target protein on total cell DAPI-positive nuclei and normalizing as a percentage relative to untreated cells (vehicle 0.02% DMSO) set at 100.

### 4.8. Statistical Analysis

Statistical analysis was performed using a two-tailed Student’s *t*-test to compare differences between two groups. A one-way analysis of variance (ANOVA) was performed for all experiments that required a comparison between three or more groups within one categorical variable, followed by Tukey’s post hoc test. For all experiments, results were considered significant at *p* < 0.05 (*; $; §; #), *p* < 0.01 (**; $$; §§; ##). Data are presented as mean ± SEM. Calculations were performed using GraphPad INSTAT 3.3 software (GraphPad, San Diego, CA, USA).

## Data Availability

Not applicable.
